# The Steps to Therapeutic Drug Monitoring: A Structured Approach Illustrated With Imatinib

**DOI:** 10.3389/fphar.2020.00177

**Published:** 2020-03-03

**Authors:** Thierry Buclin, Yann Thoma, Nicolas Widmer, Pascal André, Monia Guidi, Chantal Csajka, Laurent A. Decosterd

**Affiliations:** ^1^ Service of Clinical Pharmacology, Department of Laboratory Medicine and Pathology, Lausanne University Hospital (CHUV) and University of Lausanne (UNIL), Lausanne, Switzerland; ^2^ School of Management and Engineering Vaud (HEIG-VD), University of Applied Science Western Switzerland (HES-SO), Yverdon-les-Bains, Switzerland; ^3^ Pharmacy of Eastern Vaud Hospitals, Rennaz, Switzerland; ^4^ Institute of Pharmaceutical Sciences of Western Switzerland, University of Geneva, Geneva, Switzerland; ^5^ Center for Research and Innovation in Clinical Pharmaceutical Sciences, Institute of Pharmaceutical Sciences of Western Switzerland, Lausanne University Hospital (CHUV) and University of Lausanne (UNIL), Lausanne, Switzerland

**Keywords:** drug monitoring, molecular targeted therapies, pharmacokinetic-pharmacodynamic models, pharmacometrics, precision medicine, dosage individualization

## Abstract

Pharmacometric methods have hugely benefited from progress in analytical and computer sciences during the past decades, and play nowadays a central role in the clinical development of new medicinal drugs. It is time that these methods translate into patient care through therapeutic drug monitoring (TDM), due to become a mainstay of precision medicine no less than genomic approaches to control variability in drug response and improve the efficacy and safety of treatments. In this review, we make the case for structuring TDM development along five generic questions: 1) Is the concerned drug a candidate to TDM? 2) What is the normal range for the drug's concentration? 3) What is the therapeutic target for the drug's concentration? 4) How to adjust the dosage of the drug to drive concentrations close to target? 5) Does evidence support the usefulness of TDM for this drug? We exemplify this approach through an overview of our development of the TDM of imatinib, the very first targeted anticancer agent. We express our position that a similar story shall apply to other drugs in this class, as well as to a wide range of treatments critical for the control of various life-threatening conditions. Despite hurdles that still jeopardize progress in TDM, there is no doubt that upcoming technological advances will shape and foster many innovative therapeutic monitoring methods.

## Introduction

The methodology of clinical drug development has evolved impressively during the past few decades. In particular, companies and registration authorities have adopted high standard pharmacometric approaches and tools, enabling sophisticated analyses, modeling and simulation of pharmacokinetic (PK) and pharmacodynamic (PD) data (Csajka and Verotta, 2006). Modern pharmacometric methods can be traced back to the late sixties and owes much to the seminal contributions of Lewis Sheiner at the University of California San Francisco (Holford and Sheiner, 1982). Interestingly, his efforts started with the aim of improving patient care through *therapeutic drug monitoring* (TDM) (Sheiner et al., 1975), i.e., the measurement of circulating concentrations of a drug to adjust its dosing regimen, so as to reach a defined target exposure associated with optimal efficacy and minimal toxicity (Clarke, 2016). TDM was rather new practice at this time. It is only later that, with Stuart Beal, he derived from this early computer tool the first version of the NONMEM software, which became and still remains the reference program used for PK-PD modeling during drug development. Pharmacometrics nowadays influences all steps of pharmaceutical research, from preclinical tests through clinical phases up to drug labeling and approval (Bhattaram et al., 2005). In particular, it brings a rational support to the elaboration of dosing regimens adapted to patients' characteristics and contributes to optimize the design of pivotal Phase III trials, whose success represents the key condition for marketing approval by authorities and for uptake by prescribers. Still, once drugs are commercialized, the amount of pharmacometric knowledge accumulated during their development seems to lose most of its usefulness for patients' care, apart from some pieces of information reflected in dosage recommendations of the summary of product characteristics. There remains a significant *implementation gap* between pharmacometric research and pharmacotherapeutic practice. Despite impressive progress, Sheiner's aspiration that pharmacometrics should ultimately serve for TDM and patient care remains poorly fulfilled.

The *precision medicine* initiative, launched in the USA during the presidency of Barack Obama, aims to collect large amounts of genetic and biomedical information in a sizeable sample of healthy and sick people, to identify relevant sources of variability and take them into account in order to tailor accurately prevention and treatment strategies at the individual level (National Research Council Committee on, 2011; Collins and Varmus, 2015). Precision medicine is often presented as the next milestone of medical progress after *evidence based medicine*, which should supersede one-size-fits-all approaches restricted to the standard average patient. The proponents of precision medicine mostly think about genomics and further omics to support the personalization of treatments. They all too often neglect another important line of approaches for achieving this aim, namely, to individualize treatments based on the monitoring of drugs' exposure, effects, and disease evolution biomarkers (Cremers et al., 2016; Jang et al., 2016). Actually, TDM can relevantly benefit patients in clinical practice, through preventing or correcting both underdosing deleterious to therapeutic efficacy and overdosing leading to toxicity and subsequent treatment cessation.

While the right choice of drug doses is denoted since antiquity as a concerning issue for physicians, the modern era of dosage individualization based on the monitoring of a biological parameter began in the 1920s, with the advent of diabetes treatment with insulin, found to require a thorough follow up of either glycaemia or glycosuria (Lesko and Schmidt, 2012). In the 1950s, warfarin, initially commercialized as a rodenticide, could be turned into a human anticoagulant only thanks to prothrombin time measurement enabling its precise dosage adjustment. In the 1960s, the monitoring of blood concentrations of a few drugs with narrow therapeutic index was shown to improve their safety, and became widely available: this was the case for lithium, digoxin, phenytoin, phenobarbital, theophylline, and the aminoglycosides. Thereafter, this list integrated vancomycin, carbamazepine, cyclosporine, and tacrolimus, which still currently represent the therapeutic molecules most commonly measured in blood (Clarke, 2016). The introduction of all this TDM into clinical practice essentially followed empirical approaches, with little PK-PD support and scarce validation by randomized controlled trials. Model-based approaches advocated by visionary forerunners (Sheiner et al., 1975; Jelliffe, 1983; Lenert et al., 1989) did not meet with wide uptake until now, regarding either starting dose decisions or TDM dosage adjustments (Darwich et al., 2017). Nowadays, TDM measurements are still mostly performed in clinical chemistry labs, and the delivery of results is rarely associated with specialized pharmacological interpretation. However, recent developments in analytical technologies enable at present the measurement of an unprecedented number of drugs with excellent performances at affordable costs (Decosterd et al., 2016). In addition, recommendations for TDM have been expressed about hundreds of therapeutic agents, e.g. anticancer drugs (Widmer et al., 2014), biologics (Imamura, 2019), antiretrovirals (Punyawudho et al., 2016), anti-infectives (Muller et al., 2018), psychotropic agents (Schoretsanitis et al., 2018) etc. If such measurements are to become largely available in routine medical practice, traditional empiricism will not suffice anymore to support the clinical interpretation of drug concentration results by practitioners. Neither will specialized clinical pharmacologists be available in sufficient number to do the job for all patients receiving critical treatments that require fine-tuned dosage. In our opinion, one important bottleneck that limits until presently the development and large uptake of TDM is the lack of robust, versatile, user-friendly computer tools based on a well-structured pharmacological reasoning and operable by all types of practitioners (Fuchs et al., 2013), who will still benefit from specialized pharmacological advice when necessary. Hopefully, in parallel with relentless progress in computer sciences and widespread adoption of electronic medical records, improvements in TDM support systems are to expect soon (Kumar et al., 2019).

Our paper aims at reviewing the sequential steps involved in setting up the TDM of a given drug, through the appropriate concatenation of pieces of pharmacometric knowledge. These steps, formulated as generic questions, delineate the clinical development phases of TDM, considered in a way similar to diagnostic tests (Sackett and Haynes, 2002). The very same questions should also serve as roadmap for the computer-assisted interpretation of TDM results in clinical practice. As an illustration, we recount our contribution to the development of a TDM program for imatinib, the first targeted anticancer agent brought onto the market. We finally discuss the hurdles and the hopes that prevail today in this area.

## Five Steps to TDM

### Is the Drug a Candidate to TDM?

TDM is certainly not suitable for every drug in every patient and every disease. For a given treatment, a preliminary question thus pertains to whether TDM should enter into consideration. Pharmacological characteristics of TDM drug candidates have been devised by several authors (Spector et al., 1988; Ensom et al., 1998; Gross, 1998; Holford and Buclin, 2012) and can be summarized as follows:

1. significant *between-subject PK variability*, poorly predictable from individual patients' characteristics (such as serum creatinine for drugs excreted by renal filtration), making a standard dosage achieve a wide range of concentration levels among different patients;
2. acceptable *PK stability*, limited within-subject PK variability over time [i.e., the combination of inter-occasion variability and assay and/or model-related errors (Abrantes et al., 2019)], making a TDM measurement representative of the patient's regular exposure level;
3. consistent *PD relationships* between concentration exposure and response and/or toxicity, along with reversibility of effects following changes in exposure, enabling the delimitation of a range of concentrations associated with optimal efficacy and minimal toxicity;
4. narrow *therapeutic margin* with respect to between-subject PK variability, forbidding the use of very high standard doses in all patients to ensure overall efficacy (Holford and Buclin, 2012);
5. absence of *pharmacodynamic markers* of therapeutic response and/or toxicity readily assessable and quickly responsive to dosage changes, which would represent a preferable alternative to TDM (such as INR for coumarin anticoagulants);
6. sufficient *treatment duration* and criticality for patient's condition to justify dosage adjustment efforts.

Most of these criteria can already be checked based on product characteristics that must appear in the registration dossier, according to current requirements. Still there remains room for progress in the widespread assessment of exposure during Phase III clinical trials, and in the identification of exposure parameters best related with effects. In our opinion, the registration process of a new drug should systematically include an examination of this checklist, and the authorities should decide in accordance about the opportunity to require the evaluation of a TDM program, possibly during a dedicated Phase IV study. This checklist could also help to elaborate a list of marketed drugs to incorporate into a computer tool for TDM interpretation.

Imatinib, which we take as illustrative example, was launched in 2001 as the first targeted inhibitor of the spontaneously active tyrosine kinase BRC-ABL produced in myeloblasts after the Philadelphia chromosome mutation causing chronic myelogenous leukemia (CML). The drug quickly confirmed its excellent efficacy and tolerability, and became acclaimed as an unprecedented achievement in the war against cancer. It actually transformed CML, a malignant condition associated with a median survival of about 3 years, into a chronic condition manageable over the duration of a normal life (Gambacorti-Passerini et al., 2011). Moreover, it inaugurated a worldwide momentum for the search of similar targeted therapies against all types of cancers. Today, more than 40 such small molecule signal transduction inhibitors are commercialized as anticancer agents, and many more are in the pipelines of pharmaceutical companies.

Neither the manufacturer nor the authorities envisaged a TDM program when imatinib was brought onto the market, despite the fact that it essentially met all the above criteria, as confirmed by our initial observational population PK study (Widmer et al., 2004; Widmer et al., 2006), in line with others (Picard et al., 2007), while definite exposure-response relationships were reported (Larson et al., 2008). In 2006, a competitor appeared, namely dasatinib, as a second-line BCR-ABL tyrosine kinase inhibitor for CML patients losing response or developing intolerance to imatinib. This seems to have prompted the manufacturer of imatinib to realize that TDM could improve the persistence of patients under their drug, and to launch a campaign promoting the implementation of blood level measurement services throughout Europe [EUTOS program (Hellenbrecht, 2007)]. It aimed at encouraging prescribers to adjust imatinib dosages so as to keep plasma concentrations above a certain threshold ensuring the best chances of efficacy. However, robust evidence supporting this strategy was missing and in 2010, the American Food and Drug Administration sent the manufacturer a stern warning letter, enjoining them to stop disseminating unsubstantiated promotional information (Buclin et al., 2011). In the meantime, they had launched their own second-line agent for imatinib-resistant CML, nilotinib, losing henceforth any commercial interest to see patients staying under imatinib (Saglio et al., 2010). This story contributed regrettably to put on hold the development of TDM for anticancer signal transduction inhibitors. Still, with other authors, we remain convinced that TDM programs deserve to be considered for most small molecule targeted anticancer therapies (Gao et al., 2012; Josephs et al., 2013; Yu et al., 2014; Decosterd et al., 2015; Mould et al., 2015; Herviou et al., 2016; Mould and Hutson, 2017). Like others (Groenland et al., 2019), we are thus broadening our TDM offering for those drugs, which will surely serve the patients no less than personalization of drug choice according to tumor genetics (Venkatakrishnan et al., 2015).

### What Is the Normal Range for the Drug’s Concentration?

**Figure 1:**
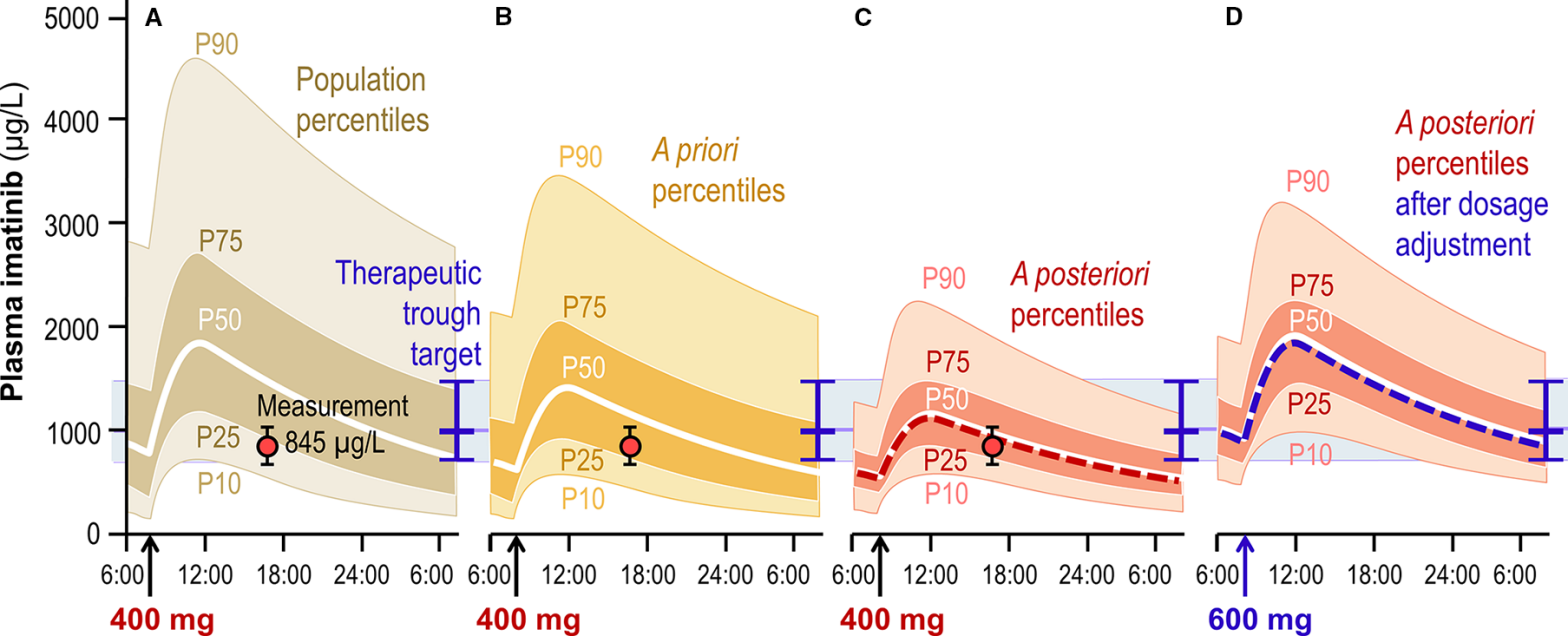
Schematic graphical representation of the interpretation of a TDM result for imatinib, measured at 845 µg/L in a 35 years, 90 kg male patient 9 hours after the last intake of his 400 mg q.d. dosing regimen. **(A)** Population percentiles showing the expected range of concentrations in the general population. **(B)** *A priori* percentiles showing concentrations expected in patients having similar individual characteristics (covariates). **(C)** *A posteriori* percentiles deduced by Bayesian inference from the *a priori* expectation and from the patient's observation (represented as the red dot, with whiskers depicting the associated intra-individual error). **(D)** *A posteriori* percentiles predicted after adjustment of the dosage to 600 mg q.d., able to drive the patient's trough concentration close to the target and the associated prediction range into the acceptance interval (represented as the blue horizontal line and band, respectively).

Launching a TDM program for a given drug firstly requires developing and validating an appropriate analytical method, which implies technical resources and expertise that we will not address here (Decosterd et al., 2016). As soon as the method is applied to clinical samples, the first question coming when facing a TDM result is about the *expectedness* of the blood levels measured: “*Is this concentration normal under the dosing regimen received by this patient with such individual characteristics?*” An answer to this question can typically be brought based on results from observational population PK studies. Such studies provide information not only on the drug's average PK parameter values, but also on their overall between-subject variability, on identified individual factors or *covariates* that explain a part of this variability, on the magnitude of the part that remains unexplained, and on the amount of intra-individual variability (sometimes split into inter-occasion and residual variability) (Sheiner and Beal, 1983; Ette and Williams, 2004b; Ette and Williams, 2004a; Ette et al., 2004). Covariates commonly include patient's sex, age, body weight, and serum creatinine, but can also comprise defined comorbidities, comedications, genetic traits, etc. When several population PK studies are available, either the “best” one can be selected based on quality criteria, or one may aggregate their results using recent techniques of model-based meta-analysis (Petit-Jean et al., 2015; Nanga et al., 2019). The quintessence of this information can be summarized graphically into *prediction percentiles* for circulating drug concentrations under a given standard dosage (**Figure 1**). A population PK model enables to deduce two types of percentiles, namely:

1. *population percentiles*, which describe the range of drug concentrations expected along the time under a given dosage across the whole target population of patients, having variable values of influential covariates;
2. *a priori percentiles*, defined for a given set of individual covariates values, which describe the range of concentrations expected specifically in a patient having these characteristics.

The variability around *a priori* percentiles is generally less than around population percentiles, as it does not include the part explained by covariates. It is still larger than the variability around *a posteriori* percentiles defined below (Section 2.4).

When interpreting an individual TDM result, obtained at a given post-dose time in a patient with known covariates values, this information about expectedness is of prime importance to evaluate the “normality” of the result, i.e. to identify if the concentration is abnormally low (e.g. in an ultrarapid metabolizer) or high (e.g. in case of troubled elimination). Obviously, abnormal concentrations may also indicate treatment adherence issues or errors in the reporting of dosing and timing information. TDM itself is not well suited to evaluate adherence, as it only reflects the actual intake of the few last doses. Adherence issues in patients are better addressed by electronic recording of medication intake (El Alili et al., 2016). On the other hand, poor adherence can represent a serious confounder in TDM results.

With regard to imatinib, besides our initial population PK study (Widmer et al., 2006), we also had access to the large database of TDM results collected in France by our colleagues of Bordeaux for the EUTOS program, which we were able to analyze (Gotta et al., 2014a). Moreover, we undertook a systematic review of other population PK studies of published to date for imatinib, which we aggregated using model-based meta-analysis (Gotta et al., 2013).

### What is the Therapeutic Target for the Drug’s Concentration?

The next crucial question raised during the interpretation of a TDM result is about the *suitability* of the concentration measured: “*Is this concentration appropriate in this patient for the condition under treatment?*” This question is about the drug's concentration-response relationships, ideally determined during population PK-PD studies, which here again should provide average estimates of relevant PD parameters together with a determination of between- and within-subject variability in PD sensitivity and possibly influential covariates. The outcome of these studies is best captured by the notion of *target concentration*, preferable to the traditional therapeutic range (Holford, 2001); yet it is convenient to define an *acceptance range* around it, with consideration to unacceptable probabilities of inefficacy or toxicity.

Actually, different types of target for concentration exposure can be defined, considering the *exposure parameter* most predictive of the therapeutic response: *trough concentration* is predominantly used, e.g. with antiepileptics, targeted anticancer agents, antivirals or betalactam antibiotics (in line with their time-dependent bactericidal activity); *peak concentration* is relevant e.g. for aminoglycoside antibiotics (concentration-dependent antibacterials); *area under curve* (AUC) or its equivalent *average concentration* are determinant for the activity and toxicity of, e.g., vancomycin or immunosuppressants; for classical cancer chemotherapies, the *cumulative AUC* is the leading PK parameter for clinical response over a treatment cycle. In addition, for certain drugs the exposure target needs to be individualized, as it is the case e.g. for vancomycin depending on type of bacteria to treat [with methicillin-resistant Staphylococci deserving higher exposure (Steinmetz et al., 2015)], for lithium with respect to the treatment phase of bipolar disorders (Goodwin et al., 2016), or for antiepileptics considering individual therapeutic responsiveness (Patsalos et al., 2008). Determination of the *minimum inhibitory concentration* (MIC, ideally corrected for plasma protein binding) of an infectious agent may bring precise information about the target to aim for. Graphically, it is straightforward to represent concentration targets and acceptance ranges overlying the percentiles curves at the appropriate time point (**Figure 1**).

Regarding imatinib, our studies mentioned above essentially confirmed the trough plasma concentration target of 1000 µg/L established previously for the treatment of CML (Larson et al., 2008). Practically, we surround it with an acceptance range of 750 – 1500 µg/L. Imatinib did also demonstrate a remarkable therapeutic efficacy against gastro-intestinal stromal tumors (GIST), a malignancy involving an activating mutation of the c-KIT tyrosine kinase, amenable to effective inhibition by this drug. Interestingly, GIST treatment seems to necessitate a dosage targeting a proper trough concentration (Demetri et al., 2009; Teng et al., 2012; Bouchet et al., 2016), possibly modulated by the tumor's genetic subtype (Widmer et al., 2008).

### How to Adjust the Dosage of the Drug to Drive Concentrations Close to Target?

Once determined the degree of expectedness and suitability associated with a given TDM result, the next question asks: “*How should the dosage be adjusted in this patient so as to reach the optimal circulating exposure target?*” This question is about the clinical exploitation of the measurement and the predictive performance of calculations derived from population PK-PD models. Bayesian inference is widely recognized as the best conceptual framework for such elaborations (Mould et al., 2016), and is implemented in various declensions in computer tools currently available (Fuchs et al., 2013; Kumar et al., 2019). The general idea is to calculate the maximum likelihood values of the patient's PK parameters by confronting the *a priori* prediction of his/her concentration curve (including the effects of influential covariates) with the information brought by the concentration measurement result. Various algorithms are available to find an optimal trade-off between both sources of knowledge, each weighted according to its respective amount of uncertainty: the *a priori* prediction comes with a certain variability (described by the percentiles defined above), while the observed concentration comes with a certain error (that lumps together biological fluctuations, model imprecision, and laboratory inaccuracy). The resulting *a posteriori* concentration curve of the patient can be graphed surrounded with *a posteriori percentiles* of prediction, which represent the expectation range for subsequent observations. When several measurements are performed in the patient, they progressively decrease the prediction uncertainty around his/her individual curve, which asymptotically reduces to the (incompressible) intra-individual variability. The computation of these percentiles is non-trivial and not widely offered by current computer tools. Eventually, various dosage adjustments are proposed, taking into account the formulation strengths available for the drug. For oral treatments, this often limits to few possibilities the range of practicable dosages. These dosage proposals are then tested for their ability to drive the patient's *a posteriori* curve sufficiently close to the therapeutic target, so as to keep the largest part of *a posteriori* percentiles inside the acceptance range (**Figure 1**). In our practice, as acceptance ranges are rather large, a suitable dosage can generally be found for not only injections or infusions, when the dose is selected in a continuum, but also oral formulations, despite the limitation in adjustment precision due to the availability of one or a few strengths.

An appreciable advantage of these calculations is that they free the clinicians from the requirement of drawing TDM blood samples exactly on the time defined for the concentration target (e.g. trough time just before next dose). For imatinib, we concretely confirmed the good predictive performance of a Bayesian inference algorithm to extrapolate trough concentrations from samples taken at random times during the dosing interval (Gotta et al., 2012). Moreover, Bayesian inference allows estimating the maximum likelihood AUC (integrated concentration exposure over time) from one or a few observation points (Rousseau and Marquet, 2002). The availability of 100- and 400-mg tablets of imatinib enables fairly flexible dosage adjustments around the 400 mg q.d. standard dosage.

Of course, the decision whether and how to alter drug dosage must still be weighted by various other clinical considerations besides TDM measurement and interpretation. Caution toward artefacts related to poor compliance, errors in dosing and sampling time, or inaccuracies of measurement might indicate to repeat TDM before changing treatment dosage. The patient's clinical condition together with available biological or imagery markers of the therapeutic response should always be taken into consideration. An old, still valid adage of TDM says: “Do not treat blood levels, treat patients!”

### Does Evidence Support the Usefulness of TDM for This Drug?

Last but not least, the ultimate question pertains to the *usefulness* of TDM: “*Will TDM and subsequent dosage adjustment improve this patient's therapeutic outcome?*” Bringing an evidence-based answer to this question requires challenging our TDM strategy during prospective randomized controlled clinical trials comparing relevant clinical outcomes. Various methodologies can be used, such as randomized assignment of TDM intervention compared to no TDM, or randomized dosage adjustment compared to no adjustment selectively in patients with low or high exposure, or comparison of clinical outcomes in a population of patients before and after introduction of TDM. No clear consensus emerges about their respective merits. The question whether TDM should be offered or not to all patients receiving a given drug is just one among others, sometimes more appropriate to address in a study. Clinical investigations may aim at identifying specific subpopulations of patients requiring TDM, considering the treated condition, particular comorbidities, comedications, demographic or genetic traits etc. The frequency of TDM controls deserves attention as well: should the drug be monitored and its dosage adjusted only once on treatment initiation? Or conversely, should the circulating concentrations be regularly checked in the patients? Alternatively, should TDM be specifically triggered by defined intercurrent events? (Glasziou and Aronson, 2008). Another question pertains to the context for TDM performance: is the prescriber's practice the best place for doing TDM? Should TDM preferably be performed in medical laboratories in charge of blood analysis? Would pharmacists be in a suitable position to take over TDM tasks? Eventually, cost-effectiveness analyses of TDM have also a definite importance.

Support is often difficult to find among drug manufacturers tending to dislike TDM, felt as an impediment complicating prescription (drug candidates that would heavily rely on TDM are at high risk to drop out of development pipelines). Very few incentives come from the authorities, the prescribers or the patients. Neither will public funders or private charities easily accept to cover the costs of TDM research, as they understandably consider not in their duties to improve the utilization of profit-generating drugs.

As we had already launched a TDM for imatinib, in 2008 the manufacturer approached us for its broad provision to patients of our country. Yet, rather than simply accepting to offer blood level testing to CML patients, we insisted on setting up a controlled clinical trial, aiming to validate the clinical usefulness of this TDM, in accordance with our own recommendations (Buclin et al., 2012). During our previous experience with antiretrovirals (Haouala et al., 2013), we did already try to launch a randomized controlled trial comparing the efficacy and tolerability of efavirenz with or without TDM-based dosage adjustment. However, our prescribing colleagues forcefully refused this project, as they deemed unethical to deprive a control group from any access to TDM. Thus, to prevent a similar refusal of a trial on imatinib TDM in CML patients, we rather opted for a comparison between *routine* TDM in all patients *versus rescue* TDM only in case of clinical problems, such as escape from control of the disease or poor tolerance (Gotta et al., 2014b). Following interruption of the manufacturer's support in 2011, the trial failed to include the pre-specified number of patients. Our study results, analyzed in intention to treat, did not reveal an overall clinical benefit for imatinib TDM. However, we realized that our results were flawed by a significant lack of compliance among the prescribers with regard to our dosage adjustment propositions in the intervention group receiving routine TDM. A *post-hoc* analysis actually indicated better outcomes in the subgroup of patients whose physicians had correctly applied our dosage suggestions (Gotta et al., 2014b). The clinical benefit of imatinib TDM for newly diagnosed CML patients received confirmation from an independent trial run in France (Rousselot et al., 2015). At present, TDM for imatinib is available to Swiss patients, like in other places in Europe; but despite its potential, it remains underused and not routinely recommended. Now that the drug has lost patent protection and that generics are available at reduced price, our health systems would have a financial advantage to keep CML or GIST patients as long as possible under this first line treatment, which systematic TDM might contribute to (Zuidema et al., 2019). On treatment initiation, TDM would be useful to check whether the standard dosage ensures sufficient concentration coverage, and to increase the dose otherwise. In case of suspicion of toxicity, TDM may help to safely reduce the dosage if it confirms exaggerated concentration exposure. Still there is uncertainty about decreasing the doses if high concentrations are found without evidence of intolerance, in particular as pseudo-elevations may result from abnormally high levels of alpha-1-glycoprotein (orosomucoid), a plasma carrier that binds a large fraction of circulating imatinib. A correction formula has been proposed if alpha-1-glycoprotein concentration is known, which we validated against the measurement of free imatinib, a more demanding option (Haouala et al., 2013). Another issue relates to the intracellular passage of imatinib, as the drug must reach the cytoplasm of malignant cells to meet the BCR-ABL tyrosine kinase. Transmembrane permeation and transport may thus introduce a further element of variability in intracellular receptors exposure for a given level of circulating concentration (Widmer et al., 2007).

## Discussion: Current Hurdles and Prospect for TDM

Our efforts about imatinib TDM have produced useful lessons. This meaningful case study shows how to regard the pharmacometric knowledge accumulated about a therapeutic agent as scientific bricks to build up a structured TDM program and to evaluate its real benefit for patients. Once this is achieved, the same pieces of knowledge should be called on to support the sequential steps of clinical TDM results interpretation. Clearly, TDM deserves consideration for most small molecule signal transduction inhibitors used by oncologists (Haouala et al., 2009; Gao et al., 2012; Widmer et al., 2014; Cardoso et al., 2018). We believe that the responsibility of requesting such evaluation for novel therapeutic agents belongs to registration authorities, as we expressed it in a recent statement of the International Society of Pharmacometrics (Maloney et al., 2018). A fair number of drugs already on the market in many therapeutic areas would benefit as well of the development of TDM programs for improving both their efficacy and tolerability.

Some significant hurdles actually explain why TDM remains difficult to promote despite its promises for patient management. Analytical methods have long been a problem. Nowadays, efficient analytical methods have become relatively easy to develop and validate, thanks to the advent of liquid chromatography coupled with mass spectrometry (Decosterd et al., 2016). Still, analytical methods demand large, remote central laboratories, hence often sample postage incurring significant delays. The moderate, yet non-negligible costs of TDM may also affect its utilization. The cost-effectiveness of TDM has infrequently been evaluated for specific drug classes or medical conditions. A single systematic review dealing with all TDM commonly practiced concluded mainly to an overall lack of appropriate cost-effectiveness analyses, while showing mostly favorable conclusions in studies addressing this aspect (Touw et al., 2005). Regarding recent targeted anticancer agents, one may conjecture that their high price would make it easy to confirm cost-effectiveness, should TDM appear able to improve only by a few percent their overall efficacy. Still evidence is lacking to date for supporting this hypothesis.

For both patients and health care providers, the constraint of standardized sampling time (usually trough) represents a problem. While computer-assisted Bayesian PK interpretation overcomes this constraint, this still requires the careful recording of current dosage, last dose intake, relevant covariate values, and actual sampling time, all pieces of information sometimes much more difficult to obtain than the sample itself. The interpretation of concentration results remains uneasy for practitioners, despite the increasing availability of software tools, still not entirely satisfactory (Fuchs et al., 2013; Kumar et al., 2019). Moreover, even when provided with appropriate assistance from a clinical pharmacologist, the prescribers are often reluctant to modify the drug dosages, because this is not yet rooted enough in the medical culture. The global unwillingness of drug companies regarding any complication to prescription prevents both the development of TDM programs and the dissemination of TDM recommendations elaborated through independent research. The resulting paucity of good quality clinical research in this area closes the loop of what might resemble a desperate vicious cycle.

Nevertheless, there are some good reasons for hope regarding the future of TDM. Analytical methods continue to progress toward better simplicity, operability, and automation, enabling, e.g., the use of alternative matrices such as saliva, interstitial fluid or dried blood spots facilitating sample collection (Gallay et al., 2018). In another direction, point-of-care TDM methods are currently being developed (Sanavio and Krol, 2015), using innovative analytical methods (Tenaglia et al., 2018). Further aspects of research in TDM also follow an ascending curve, with an increasing number of scientists from various disciplines, notably biomedical engineers, discovering this vast field of investigation. Not only drug concentrations but also a variety of efficacy and tolerability biomarkers will enable therapeutic monitoring in its broad sense to become a mainstay of tomorrow's precision medicine. Progress in medical information technology will soon lead our health care systems to a fully connected state, thus solving difficulties related to the communication and medical utilization of concentration measurements. Pharmacological interpretation of TDM will benefit from incoming computer tools of improved user-friendliness and performances, whose development is underway (Dubovitskaya et al., 2017; Tucker, 2017). Young generations of practitioners, trained from childhood on game consoles, will probably be more receptive than their seniors to precise steering of therapies with connected instruments. However, adequate therapeutic follow up will always go beyond the mere interpretation of TDM results, even supported by the best PK-PD models, and take into account all relevant aspects of the patient's condition. We thus advocate computer-*assisted* TDM by practitioners rather than automatized TDM performed by a computer. The development, dissemination, and supervision of TDM will continue to require expert clinical pharmacologists, ready to be consulted in problematic cases. On the other hand, the automatic acquisition of monitoring data will build up sizeable datasets ready for exciting novel forms of medical research (Horwitz et al., 2017). Finally, the global move toward patient empowerment, facilitated by appropriate portable applications, will stimulate the active involvement of patients in their own therapeutic monitoring. Most patients will certainly enjoy to visualize of circulating exposure incurred by their medicinal drugs, and an increasing number of them are keen to take the helm of self-monitoring.

## Author Contributions

This narrative review develops a topic presented by TB during the PAGE meeting of 2018 in Montreux, Switzerland. TB conceived the paper. TB, NW, CC, MG, and LD conducted the research about imatinib TDM. YT conceived the software implementation of TDM steps. TB, NW, PA, and LD are responsible for TDM operations in our institution. Authors approve present publication and declare that no other manuscript describing essentially the same contents has been submitted or published elsewhere.

## Funding

Some of the research summarized in this article has received specific grants from Novartis Inc., Basel, Switzerland, and from the NanoTera initiative of the Swiss National Science Foundation (projects ISyPeM 1 and 2, Innovative Systems for Personalized Medicine). The funder was not involved in the study design, collection, analysis, interpretation, and publication of the data (Gotta et al., 2014b). Neither did this funder intervene during the writing of the present article or the decision to submit it for publication.

## Conflict of Interest

TB, YT, CC, and LD are involved in the development of the TUCUXI software tool for the support of TDM, aiming towards commercialization.

The remaining authors declare that the research was conducted in the absence of any commercial or financial relationships that could be construed as a potential conflict of interest.
